# Aging microglia: old friends greet new enemies

**DOI:** 10.18632/aging.204317

**Published:** 2022-09-28

**Authors:** Yifei Dong, V. Wee Yong

**Affiliations:** 1 Hotchkiss Brain Institute and the Department of Clinical Neuroscience, University of Calgary, Calgary, Alberta, Canada

**Keywords:** multiple sclerosis, neurodegeneration, osteopontin, oxidized phosphatidylcholines

Aging associates with the onset and progression of neurodegenerative diseases in the central nervous system (CNS), including multiple sclerosis (MS). Notably, individuals with relapse-remitting MS convert to non-remitting secondary progressive MS with higher frequency as they age. Existing therapeutics often fail to prevent long-term neurodegeneration and disability in MS, partially because the mechanisms that drive neural injury are not fully understood. Thus, to develop better therapeutics for progressive MS, it is important to determine what mediates neurodegeneration and how risk factors such as aging accelerate deterioration. Recent single cell RNA sequencing (scRNAseq) studies report prevalent age-associated transcriptomic changes in CNS cells including astrocytes, oligodendrocytes, and microglia [1,2]. Further investigations on how aging impacts the biology of CNS cells and their ability to respond to mediators of neurodegeneration will help to identify new therapeutic strategies.

Recently, we identified oxidized phosphatidylcholines (OxPCs) as potent neurotoxic molecules that require clearance by microglia in the spinal cord of mice [3]. OxPCs form when normal phosphatidylcholines of lipid components of plasma membrane and myelin sheaths undergo peroxidation. This process often occurs when free radicals are released during inflammation and tissue injury. Since excess OxPCs can promote cell death and tissue pathology through multiple mechanisms, the detection of OxPC accumulation in the CNS in MS, frontotemporal dementia, amyloid lateral sclerosis, as well as spinal cord injury suggests these molecules may be common mediators of neurodegeneration relevant to multiple neurological diseases [4]. Moreover, as microglia are the predominant immune cells that neutralize OxPCs in the CNS [3], determining how aging may alter their neuroprotective functions and whether this exacerbates neurodegeneration could provide clues to understand why neurological diseases progresses with age. To tackle this question, we first compared OxPC induced lesions in the spinal cords of young (6-week-old) and aging (52-week-old) mice and found that aging lesions suffer greater tissue and axonal loss [5]. While fewer microglia/macrophages accumulated in aging lesions, they had higher elevation of IL-1β and inducible nitric oxide synthase, suggesting their response to OxPCs was exaggerated and more pro-inflammatory. Using scRNAseq and spatial RNAseq, we compared the transcriptomes of young and aging microglia/macrophages and identified numerous differentially expressed genes altered by either aging, OxPC, or both aging and OxPC. In response to OxPCs, highly upregulated genes in microglia/macrophages include *Apoc1*, *Cstd*, *Spp1*, *Lpl*, *Lgals1*, *Gpnmb*, *Anxa2*, *Lyz2*, and *Lipa*; these genes are also elevated in microglia/macrophages enriched in MS lesions [6]. These observations together with the detection of OxPC accumulation in MS lesions suggest that microglia/macrophage exposure and response to OxPC neurotoxicity also occurs in MS and this may be further studied using the stereotactic OxPC injection model we have established in mice [3,5].

**Figure 1:**
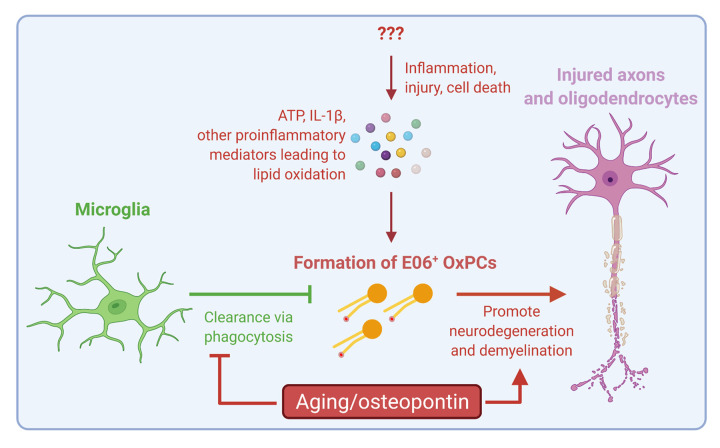
Summary of recent findings in [3,5]. While it is unclear what are the initial inducer(s) of inflammation, injury or cell death in MS and other neurological diseases, release of alarmins such as ATP or IL-1β in the CNS promotes an inflammatory environment that leads to OxPC deposition. Excess OxPCs are neurotoxic and drive demyelination and neurodegeneration. Microglia are the predominant immune cells of the CNS responding to OxPCs; they are normally neuroprotective and help neutralize OxPCs by phagocytic clearance. In the aging CNS, microglia have significantly altered transcriptome and functions, including an upregulation of the gene *Spp1*, which encodes for osteopontin. Aging microglia and the overexpression of osteopontin in the aging CNS environment leads to a defective response against OxPCs, resulting in exacerbated neurodegeneration. Figure created at Biorender.com.

Consistent with other reports, we also found aging reprograms microglia/macrophage to become more pro-inflammatory, particularly in the upregulation of interferon-response associated genes [1,2,5]. More importantly, the defective microglia/macrophages in aging OxPC lesions further elevated transcripts including *Cd74*, *H2-*Aa, *Fth1*, *Ftl1*, and *Spp1*, which are also enriched in microglia/macrophages found in the chronically inflamed edge of slowly expanding MS brain lesions that associate with disease progression [7]. This observation suggests aging and/or chronic inflammation polarize normally neuroprotective microglia to become more pro-inflammatory and potentially harmful when ‘greeting new enemies’ at sites of CNS injury. Given *Spp1*, which encodes the glycoprotein osteopontin, is reported as a highly age-upregulated and MS-elevated gene in multiple scRNAseq studies [1,2,6,7], we hypothesized its upregulation by aging microglia/macrophages worsens OxPC neurotoxicity. In support of this hypothesis, osteopontin converted perinatal microglia into an aging associated phenotype where pro-inflammatory cytokines including *Il1b* and *Nos2* as well as multiple interferon-associated genes are induced. In addition, we observed that co-injection of osteopontin and OxPCs increased neurodegeneration over OxPC injection alone in young and aging mice, whereas osteopontin knockdown using adeno-associated virus expressing *Spp1*-targeted shRNA reduced OxPC injury in aging mice. Thus, aging-upregulated osteopontin promotes a defective microglia response and contributes to the exacerbation of OxPC neurotoxicity in the aging CNS (summarized in Figure 1) [5].

While these results implicate osteopontin as a promising therapeutic target for neurodegeneration and MS progression, additional investigations are needed to determine its signaling mechanisms in the CNS. Moreover, given osteopontin has pleiotropic functions including tissue remodeling and repair, it will be important to compare its spatial and temporal activity, as well as downstream targets, in the healthy-aging versus diseased-aging CNS. As aging also induces numerous other transcriptomic changes in microglia, systematic analyses are required to assess which are compensatory mechanisms against aging and which alterations cause these cells to lose their normal functions. The detection of a greater number of differentially expressed genes in aging microglia during OxPC injury compared to aging microglia at steady state suggest that the impact of aging becomes more pronounced during disease, highlighting a need to target microglia and restore their neuroprotective functions during MS pathology and progression in aging individuals. However, as monocyte-derived macrophages also increase during aging and neurodegeneration [8], future studies should compare their behaviour versus that of microglia in aging and in various diseases including MS, such as in their responses to OxPCs. Evaluating their functional differences will inform on whether there is a need to specifically target each population to reduce pathology or to promote repair.

Collectively, aging profoundly reprograms microglia transcriptomes and subverts their neuroprotective functions. Thus, therapeutic rejuvenation of senescent microglia may help slow or stop neurodegenerative changes in the aging CNS.
